# Impacts of the COVID-19 on all aircraft emissions of international routes in South America

**DOI:** 10.1016/j.isci.2022.104865

**Published:** 2022-08-06

**Authors:** Qiang Cui, Yilin Lei, Ye Li, Peter F. Wanke

**Affiliations:** 1 School of Economics and Management, Southeast University, Nanjing 211189, China; 2 School of Business Administration, Nanjing University of Finance and Economics, Nanjing 210023, China; 3 COPPEAD Graduate Business School, Federal University of Rio de Janeiro, Rio de Janeiro 999074, Brazil

**Keywords:** Atmospheric science, Atmospheric observation, Business, Decision science

## Abstract

The COVID-19 pandemic has had a significant impact on South America’s economic development, as well as its international civil aviation industry. This paper seeks to calculate the emissions of six pollutions (CO_2_, CO, HC, NOx, SO_2_, and PM2.5) from the international routes in South America during 2019–2021 and discusses the impacts of COVID-19 on the emission change. The modified BFFM2-FOA-FPM method is proposed to unify the CO_2_ and non-CO_2_ calculations. The calculated results' average error rate is about 5.12%. The results showed that COVID-19 affected all emissions, including the number of routes, average flight distance, aircraft configuration, the proportion of CCD phase emissions, average emissions, etc. In addition, some airlines increased the number of flights and aircraft types during the pandemic, increasing emissions. The results give a reasonable data basis for the aviation industry in South America to formulate emission reduction policies.

## Highlights

• Calculated the overall emissions of the international routes in South America
• The modified BFFM2-FOA-FPM method is proposed to unify the CO_2_ and non-CO_2_ calculations
• Affected by the epidemic, some regional aircrafts were widely used in 2020
• The average emissions of the routes and airlines decreased significantly

## Graphical abstract

**Figure:**
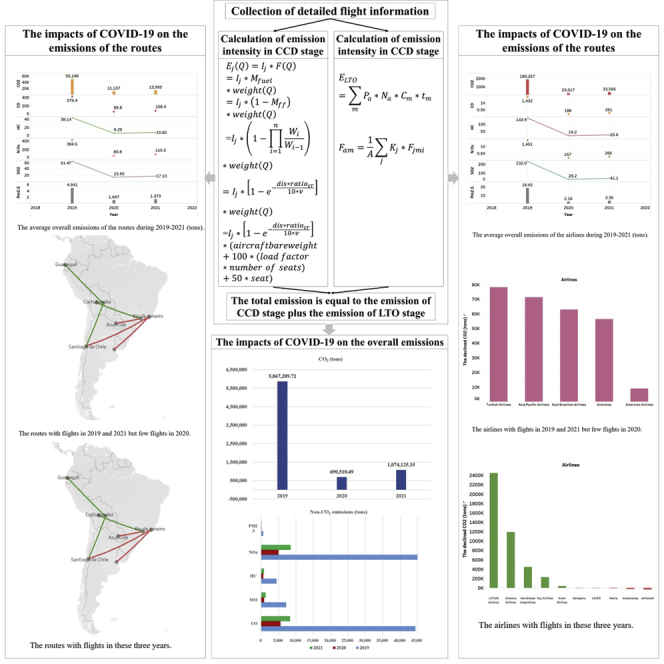

## Introduction

The rapid growth of air travel has led to the intensification of climate impacts. The main factors that affect climate change, such as contrails, fuel consumption, aviation emissions, etc., have attracted more and more attention (Xue et al., 2020). However, COVID-19 has severely impacted the world economy and greatly restricted people’s transportation, thus causing significant disruption to the development of the civil aviation industry. The epidemic’s impact on different countries and regions varies significantly due to various prevention measures and vaccination practices. South America has become the first continent globally to have cases in every country in 2020, with many leaders infected with COVID-19, such as President of Argentina Fernandez and President of Brazil Bolsonaro (González-Bustamante, 2021). Therefore, it is of great interest to study the impact of COVID-19 on aviation in South America.

To coordinate the world civil aviation affairs and formulate international standards of aviation statistical analysis, the International Civil Aviation Organization (ICAO) has divided the world into six economic statistical areas: North America, Europe, Asia Pacific, the Middle East, South America, and Africa. As South America is an essential part of the international civil aviation industry, it is vital to study the impact of COVID-19 on its aviation industry. A lot of research has focused on the effects of COVID-19 on the aviation industry, mainly in four aspects: the significant losses resulting from COVID-19 (Lacus et al., 2020), restoration of passenger confidence (Lamb et al., 2020), the support of the government and relevant institutions (Czerny et al., 2021), self-rescue measures of civil aviation enterprises (Samanci et al., 2021), and the recovery path (Cui et al., 2022a, 2022b). However, there has been little research into the impact of COVID-19 on aviation emissions.

Carbon dioxide (CO_2_) accounts for most aircraft emissions, dramatically impacting the greenhouse gas effect. As a result, some studies have focused on calculating CO_2_ emissions. For example, through ICAO Carbon Emissions Calculator, Larsson et al. calculated the GHG emissions from international air travel in Sweden between 1990 and 2014 (Larsson et al., 2018). In addition, Kito et al. applied a decomposition analysis to account for the CO_2_ emissions of Japan’s two major airlines. As a result, they concluded that more Boeing 787 led to CO_2_ emission reductions of 1.3 million tons by the two companies (Kito et al., 2020). In addition to CO_2_, aircraft activities also generate other emissions, including carbon monoxide (CO), hydrocarbon (HC), nitrogen oxides (NOx), sulfur dioxide (SO_2_), and particulate matter (PM2.5). CO can cause significant damage to the body’s physical and mental systems near airports, and even high concentrations of CO can even cause death (Safarianzengir et al., 2020). HC and NOx may form photochemical smog, which will damage human and animal health and affect plant growth around the airports (Rice et al., 2018). SO_2_ is an integral part of acid rain, which can cause severe damage to soil, water sources, forests, etc. (Zhou et al., 2019a, 2019b). PM2.5 affects human lungs and shortens human life span (Ramírez et al., 2020).

The primary calculation methods of non-CO_2_ emissions are the ICAO method (Boeing Fuel Flow Method 2 [BFFM2] and First Order Approximation [FOA] method) (Fuglestvedt et al., 2010; Wasiuk et al., 2016), the American EPA method (Yang et al., 2018), and European EMEP method (Park and O’Kelly, 2014). Some studies have applied it to calculate non-CO_2_ emissions. For example, Ekici et al. estimated the HC, CO, and NOx emissions of the five busiest airports in Turkey during the LTO period based on the emission factors of the Turkish National Airport Authority and ICAO engine data tables (Ekici et al., 2013). Akdeniz also applied the standard ICAO method to estimate the engine exhaust emissions of the aircraft at the LTO stage at the airports (Akdeniz, 2021). The ICAO method was also used at Tbilisi International Airport in Georgia (Tokuslu, 2020), Hasan Polatkan Airport (Atasoy et al., 2021), and Chinese airports (Zhou et al., 2019a, 2019b). However, these methods are more suitable for accounting for aircraft emissions during the LTO stage and cannot be combined with CO_2_ calculations. For example, the main purpose of the EPA method is to calculate the emission inventory during the LTO cycle. EMEP calculation method mainly analyzes the emission characteristics of aero-engine from the fuel perspective and ignores the differences between engine types. The ICAO method is more general, but the distance difference is not enough, and there is no difference between specific aircraft (Cui et al., 2022a, 2022b).

Generally, the flight process consists of seven steps: Engine Starting, Taxiing, Taking Off, Climbing, Cruising, Descending, and Landing (Cui, 2019). It is usually divided into the Landing and Take-Off (LTO) cycle and the Climbing/Cruising/Descending (CCD) stage. Therefore, the overall emissions include LTO emissions and CCD emissions. This study takes the international routes in South America as an example. It calculates all the aircraft emissions during 2019–2021 to explore the impacts of the COVID-19, containing CO_2_, SO_2_, HC, CO, NOx, and PM 2.5. The data for 2019 can represent the situation before the COVID-19, and those for 2020 and 2021 represent the situation after COVID-19. Therefore, the overall emissions include the CCD emissions and LTO emissions. The emissions in the CCD stage are calculated through the modified BFFM2-FOA-FPM method. The LTO emissions are calculated based on the ICAO standard method.

The modified BFFM2-FOA-FPM method can unify the calculation of CO_2_ and non-CO_2_ emissions in the CCD stage. And this method considers the emission intensity of aircraft types, divides the route distance into several groups, and obtains the carbon emissions of aircraft types within these different distances to ensure the accuracy of the calculation results. Therefore, compared with existing studies, our results cover more detailed aircraft types, and we have modified the results according to the actual flight time to make them more realistic. The main improvements are as follows: first, we combine the BFFM2-FOA method and the Fuel Percentage Method (FPM) (Cui, 2019) to propose a new method—modified BFFM2-FOA-FPM—to calculate the emissions of the CCD stage. It can calculate CO_2_ and non-CO_2_ emissions simultaneously. Second, we divide the distances into 11 segments: 0–500 km; 501–1,000 km; 1,001–1,500 km; 1,501–2,000 km; 2,001–2,500 km; 2,501–3,000 km; 3,501–3,500 km; 3,501–4,000 km; 4,001–4,500 km; 4,501–5,000 km; and 5,001–5,500 km. Then the emission intensity of the six pollutions at different distances is calculated separately based on the modified BFFM2-FOA-FPM method. Third, we consider the specific aircraft types. For example, the A320 series has many series, such as A320–100 and A320-200. According to the aircraft information of VariFlight (2022), the main engine of the A320-100 series is V2500. Still, the A320-200 series’ engine is CFM56–5 or V2500, which may cause these two aircraft to differ in emissions significantly. In this paper, we are accurate to the subtypes. Fourth, based on the First Order Approximation (FOA) method, we calculate the emissions of PM2.5, which existing studies have not calculated.

## Results

### Statistical characteristics of the international routes in South America

**Figure 1:**
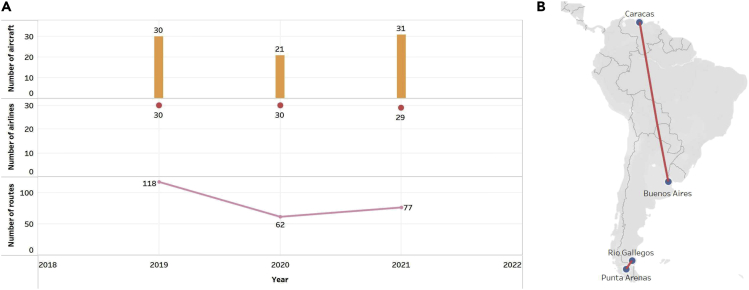
Statistical characteristics of the air routes (A) Number of airlines, number of aircraft, and number of routes. (B) The farthest route and the nearest route.

This article collects information on all the international routes in South America, and the detailed statistical features are shown in Figure 1. There are thirteen countries in South America: Colombia, Venezuela, Guyana, Surinam, Ecuador, Peru, Brazil, Bolivia, Chile, Paraguay, Uruguay, Argentina, and French Guiana.

It can be seen from Figure 1 that the international aviation industry in South America has been dramatically affected by COVID-19. The number of routes, the number of airlines, and the number of aircraft types have all declined in the wake of COVID-19. Among them, the number of routes dropped the most, directly from 118 in 2019 to 62 in 2020, a drop of 47.5%, before recovering to 77 in 2021. This result indicates that COVID-19 has significantly impacted South America’s international routes, with only a partial recovery in 2021. The longest route is from Caracas to Buenos Aires, 5,124 km; the shortest route is 187 km from Punta Arenas to Rio Gallegos. In terms of average flight distance, the average flight distance of all airlines was 2,032.69 km in 2019 but decreased to 1,932.3 km in 2020 due to the impact of COVID-19.

### The impact of COVID-19 on aircraft configuration

We summarized the frequency of each aircraft type from 2019 to 2021 and found that 320-214 was the most frequently used type in all three years. Because the distance of international airlines in South America ranges from 187 km to 5124 km, 320-214 is suitable for applying these short-haul or medium-long haul routes. However, there is a big difference between the second and third most frequent aircraft types. In 2019, the second and third place types were the 737-800 and 320-232, respectively, but they changed to the E190 and CRJ200 in 2020 and the 737-800 and E190 in 2021. This result shows that after being affected by the epidemic, regional aircrafts such as E190 and CRJ200 are widely used on international routes in South America. Although the 737-800 and 320-232 have higher passenger capacity than E190 and CRJ200, their fuel consumption per flight is also high. For example, the passenger capacity of 320-232 is about 150–180 people. Its fuel consumption per hour is about 2.5 tons; the passenger capacity of E190 is about 98–114 people, but its fuel consumption per hour is about 1.97 tons. Therefore, under the influence of COVID-19 and the unavailability of passenger rates, arranging more E190 is more cost-saving than 320-232.

### The impacts of COVID-19 on the emission intensity of the aircraft in the CCD stage

As mentioned earlier, we divide the distance into 11 segments based on the distance range: 0–500 km; 501–1,000 km; 1,001–1,500 km; 1,501–2,000 km; 2,001–2,500 km; 2,501–3,000 km; 3,501–3,500 km; 3,501–4,000 km; 4,001–4,500 km; 4,501–5,000 km; and 5,001–5,500 km. Furthermore, we considered the difference between sub-series, such as 320-214 and 320-232. Then, we get the aircrafts’ emission intensity of the six pollutions from 2019 to 2021 based on the modified BFFM2-FOA-FPM Method.

**Figure 2:**
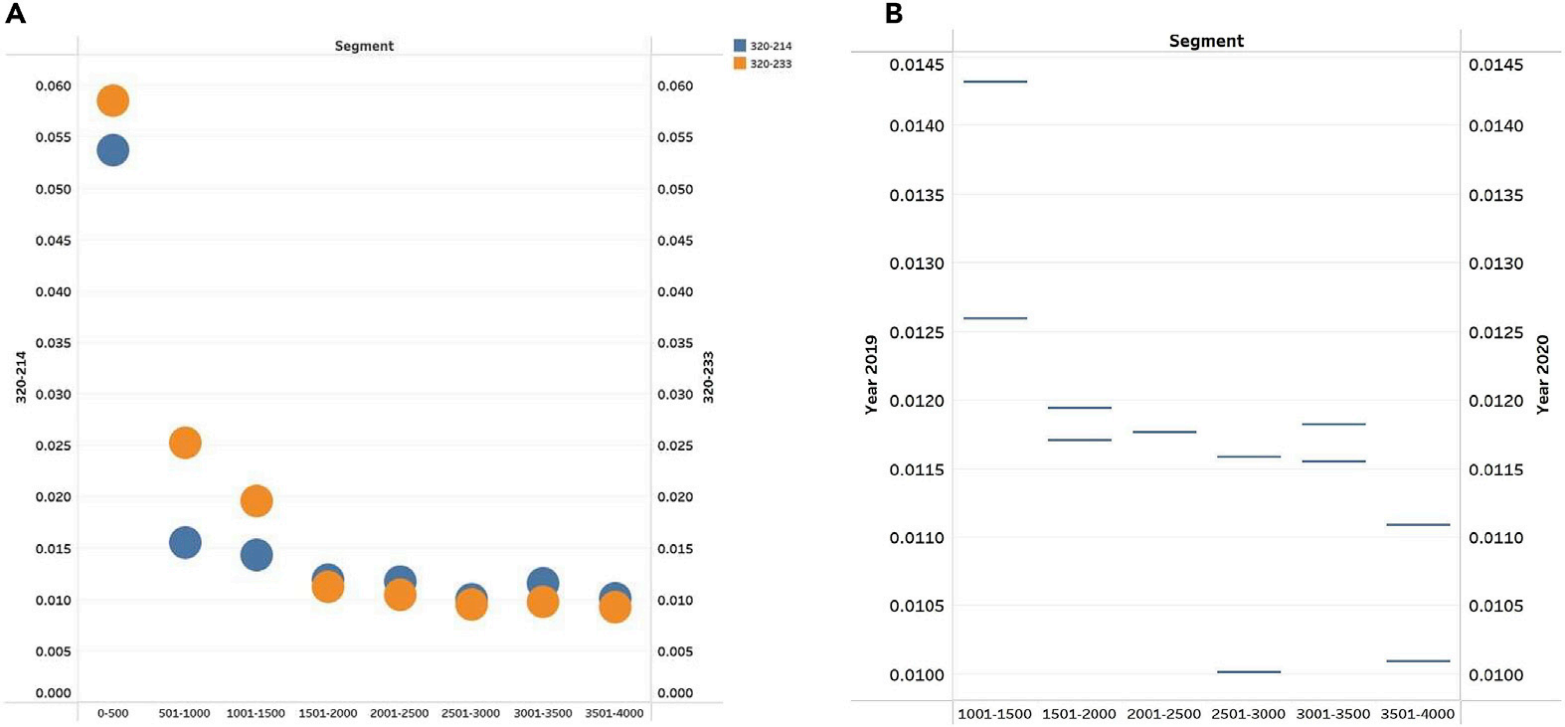
CO_2_ emission intensity of the aircrafts (ton/km) (A) Comparison of 320-214 and 320-233 in 2019. (B) Comparison of 320-214 between 2019 and 2020.

First, we analyze the difference in emission intensity of different aircraft types in the same series. Then, we consider the difference in emission intensity of subtypes compared with previous studies. As stated in Section 1, 320-214 and 320-233 are the subseries of the A320 series, so we take their CO2 emission intensities in 2019 as an example to analyze their difference, as shown in Figure 2A. In 2019, the flying distance of 320-214 and 320-233 covered 0–4,000 km. As shown in Figure 2A, the carbon emission intensities of 320-214 are less than 320-233 in the distance of 0–1,500 km but more significant than 320-233 in 1,501–4,000 km. The engines of 320-214 and 320-233 are both CFM56-5/V2500, so this result may have something to do with the actual flight route. We analyzed the flight time per unit distance of 320-214 and 320-233 in 2019 and found that in 0–1,500 km distance, the flight time per km of 320-214 is 0.00129 h/km, while that of 320–233 is 0.00159 h/km. However, in the 1501–4000km segment, the flight time per kilometer of 320-233 is 0.00118 h/km, whereas that of 320-214 is 0.00125 h/km. A longer flight time per kilometer means a more significant fuel consumption per kilometer and a larger emission intensity.

Second, we discuss the impacts of COVID-19 on the emission intensity of aircraft types. As noted earlier, the 320-214 was the most frequently flown aircraft every year from 2019 to 2021, so we selected the difference in CO_2_ emission intensity of 320-214 aircraft in 2019 and 2020 to compare and analyze the impact of COVID-19. 320-214 covers 1,001–4,000 km in 2019 and 2020, so this distance is selected for analysis, as shown in Figure 2B. We can conclude from Figure 2B that 320-214’s emission intensities in 2019 are more significant than in 2020 in the 1,001–2,500 km segment but less than in the year 2020 in the part of 2,501–4,000 km. This result is closely related to the impact of the COVID-19. Due to the COVID-19 pandemic, many flights have been canceled, air traffic control has been reduced, and the probability of delay has decreased. Therefore, the average carbon emission intensity of 320-214 in the distance of 1,001–2,500 km in 2020 has decreased. However, for medium- and long-haul flights, the impact of delays is relatively small, and the service life of 320-214 aircraft will be increased by one year in 2020 compared with 2019, so its carbon emission intensities in 2020 in the segment of 2,501–4,000 km are more prominent than 2019.

### The impacts of COVID-19 on the overall emissions

This study divides the overall emissions into LTO emissions and CCD emissions. The CCD emissions are calculated through the modified BFFM2-FOA-FPM method, and the LTO emissions are calculated based on the ICAO standard method (the detailed forms can be found in “STAR Methods”). First, we need to check the accuracy of the results in this paper. Unfortunately, we do not have data on turnover and emissions per unit for international routes in South America. Therefore, we collected the flight frequency, weight, and flight mileage of various aircraft types in 2019 to comprehensively calculate the total turnover of international airlines in South America 2019, which is about 5,871,759,805.56 ton-km. According to the relevant data from the Civil Aviation Administration of China (CAAC, 2022), the fuel consumption per ton-km is about 0.29–0.32 kg/ton-km. Multiply by the carbon dioxide emission coefficient of 3.157, and we can get an estimated 5,375,772.26 tons–5,931,886.63 tons of carbon dioxide emissions in 2019. Compared with the calculation result in this paper (5,867,289.72 tons), the error rate is about 1.09%–9.14%. Considering the statistical data of various airlines may also appear to be errors, the calculation results of this paper are relatively accurate.

**Figure 3:**
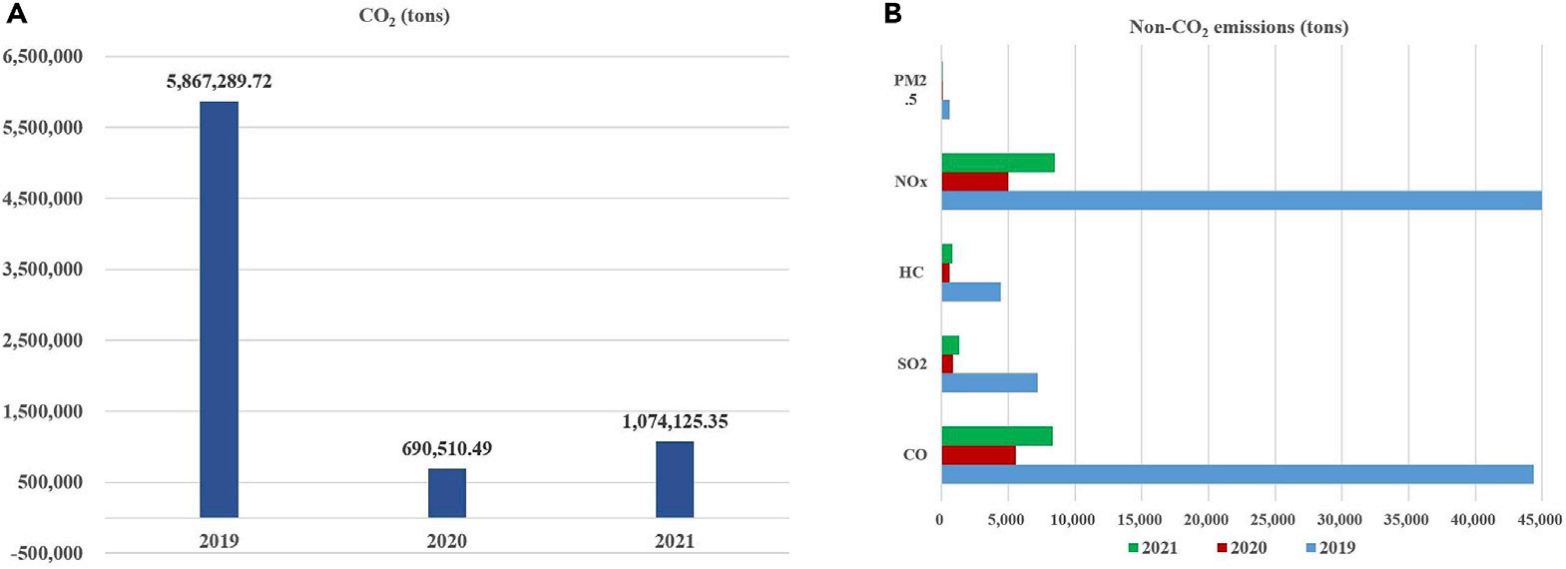
The overall emissions of the six pollutions during 2019–2021 (A) CO_2_ emissions. (B) Non-CO_2_ emissions.

The primary emissions include CO_2_, CO, HC, NOx, SO_2_, and PM2.5. The overall emissions are shown in Figure 3. Figure 3A shows CO2 emissions, and Figure 3B reveals the non-CO2 emissions. As shown in Figure 3, affected by the COVID-19, the overall emission in 2020 decreased significantly compared with that in 2019, increased slightly in 2021, but did not return to the emission before the epidemic. Taking CO2 as an example, compared with 2019, the emissions in 2020 decreased by 88.23%, and the emissions in 2021 accounted for only 18.3% of the emissions in 2019; this shows that the overall emissions have also reduced with the reduction of flights caused by the COVID-19.

Comparing Figures 3A and 3B, we can also find that the proportion of carbon dioxide in all kinds of pollution is the largest, and the balance is much higher than that of other pollution. For example, the total carbon dioxide emission in 2019 was 5,867,289.72 tons, far exceeding the second-ranked gas NOx (44,985.09 tons). In addition to CO_2_, CO and NOx are also relatively large. However, the proportion of non-CO_2_ pollution varies in different years. For example, in 2019 and 2021, the amounts of NOx are a little more significant than CO, but the latter exceeds the former in 2020. This result is related to the adjustment of aircraft configuration under the epidemic situation. As stated earlier, regional aircraft such as E190 was widely used on international routes in 2020 to replace the 320-232 commonly used in 2019. Therefore, the CO emission intensity of E190 is higher than that of 320-232. For example, in the distance segment 1,001–2,000 km, the CO emission intensity of E190 is about 1.27E-04 tons/km, whereas that of 320-232 is about 8.85E-05 tons/km. This change in aircraft configuration affected by the COVID-19 leads to more significant CO emissions than NOx in 2020.

**Figure 4:**
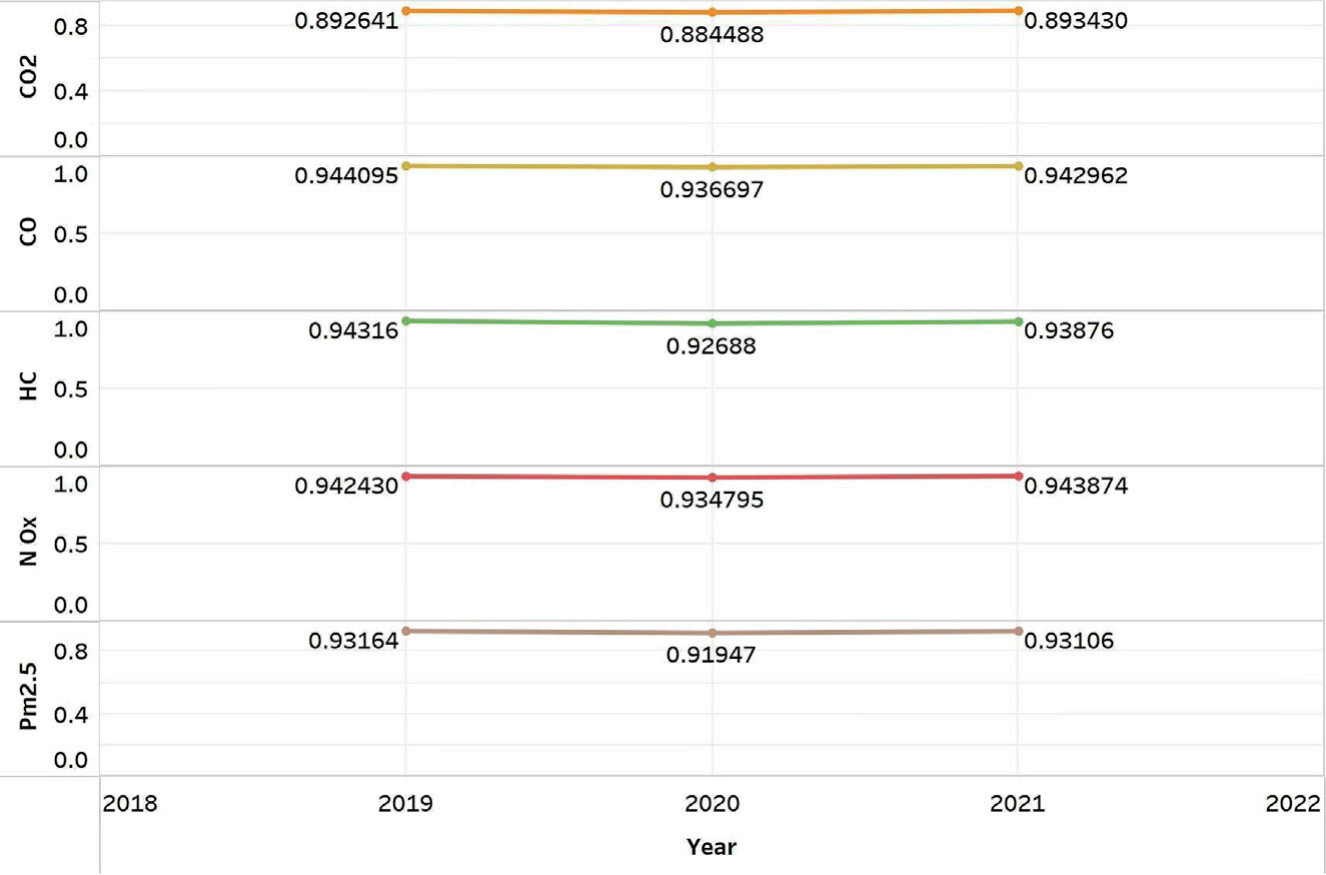
The proportions of CCD emissions in overall emissions during 2019–2021

Next, we will discuss the proportion of CCD and LTO emissions in the overall emissions. Because SO_2_ and CO_2_ change in the same ratio, we will only discuss five pollutions other than SO_2_. The detailed proportions of CCD emissions in overall emissions are shown in Figure 4.

As shown in Figure 4, affected by COVID-19, CCD emissions accounted for the lowest proportion of total emissions in 2020, related to the average flight distance. The average flight distance in 2019 was about 2,036.63 km, but it declined to 1,932.32 km in 2020. LTO emissions are not related to flight distance, but CCD is directly related to flight distance. Short average flight distance means that the proportion of LTO emission in the overall emission increases.

### The impacts of COVID-19 on the emissions of the routes

**Figure 5:**
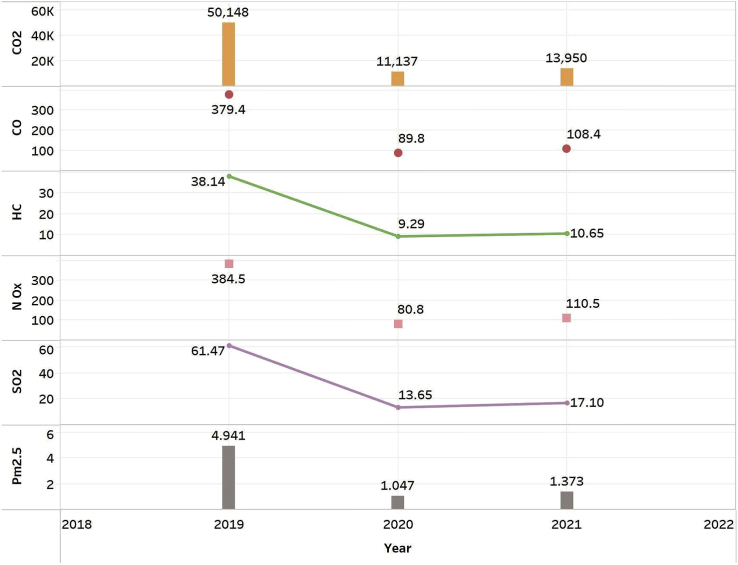
The average overall emissions of the routes during 2019–2021 (tons)

First, we will discuss the effects of COVID-19 on the average emission of the routes. The change in average overall emissions of the routes during 2019–2021 is shown in Figure 5.

As shown in Figure 5, affected by the COVID-19, the overall emissions of six pollutions decreased significantly, and the average emissions of all routes decreased significantly. The decline rates of CO_2_, CO, HC, NOx, SO_2_, and PM2.5 from 2019 to 2020 are 77.79%, 76.33%, 75.65%, 78.99%, 77.79%, and 78.80%, respectively. Furthermore, there is still a big gap between 2021 and the level before the COVID-19. The average overall emissions of CO_2_, CO, HC, NOx, SO_2_, and PM2.5 in 2021 only account for about 27.82%, 28.58%, 27.91%, 28.73%, 27.82%, and 27.78%, respectively. From these data, it can be concluded that in 2021, the international routes in South America only recovered to less than 30% before the epidemic.

**Figure 6:**
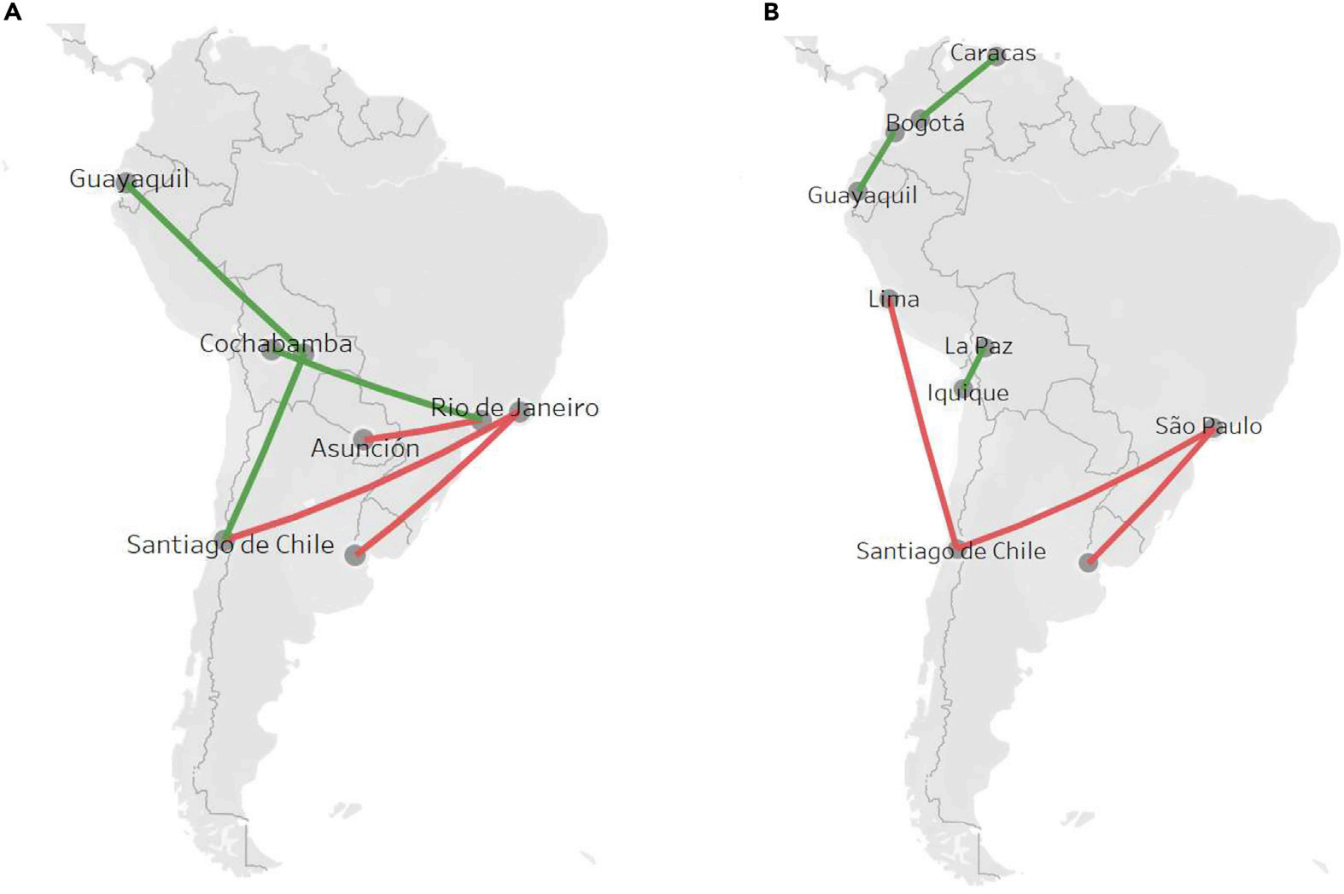
The routes with the largest decline and the routes with the least decline (A) The routes with flights in 2019 and 2021 but few flights in 2020. (B) The routes with flights in these three years.

Next, we will analyze the impacts of the COVID-19 on specific routes. By analyzing the routes from 2019 to 2021, we divide the routes into two categories: the routes with flights in 2019 and 2021 but few flights in 2020 and the routes with flights in these three years. For the former, we summarize the three routes with the most significant emissions and the three routes with the most negligible emissions in 2019, as shown in Figure 6A. For the latter, we summarize the three routes with the most significant decline and the three with the most negligible drop in 2020 compared with 2019, as shown in Figure 6B. The red ones are the routes with the most significant reduction, and the green ones have the slightest decline.

Figure 6A shows that the three routes with the most significant decline are Rio de Janeiro-Santiago de Chile, Rio de Janeiro-Buenos Aires, and São Paulo-Asunción. These three routes link to Brazilian cities, illustrating how the COVID-19 outbreak in Brazil has impacted its international civil aviation industry. In 2020, more than 7.6 million people were diagnosed with COVID-19, and the cumulative death toll was close to 200,000 in Brazil. The outbreak’s severity has led Brazil to impose strict entry-exit measures and hit its international aviation industry hard. In 2020, Brazil issued several announcements that severely restricted or banned foreigners from entering the country (Vale et al., 2021). Therefore, there were almost no flights on the three routes linking major cities in South America.

In Figure 6B, the routes with the least decline are Cali-Guayaquil, Bogotá-Caracas, and La Paz-Iquique. Notably, emissions from these routes are higher in 2020 than in 2019. The reasons for this result are different for the three routes. For Cali-Guayaquil and La Paz-Iquique, the main reason is the change in the aircraft types, but for Bogotá-Caracas, the main reason is the increase in flight frequency. For Cali-Guayaquil, the primary aircraft in 2019 are 320-214 and 319-113, but they will become 340-200 in 2020. Therefore, the average emissions per kilometer and an LTO cycle of 340-200 are much higher than 320-214 and 319-113. For example, the CO2 emission intensity in the CCD stage of 340-200 in the 501–1,000 km segment (the distance of Cali-Guayaquil is 742 km) is about 0.062 tons/km, whereas those of 320-214 and 319-113 are 0.0126 and 0.0119 tons/km. The CO2 emission in an LTO cycle of 340-200 is about 5.96 tons, whereas that of 320-214 and 319-113 are 2.79 tons. For La Paz-Iquique, the main aircraft in 2019 was CRJ200, but it became E190 in 2020. Again, the average emissions per kilometer and in an LTO cycle of E190 are much higher than CRJ200. For example, the CO2 emission intensity in the CCD stage of E190 in the 0–500 km segment (the distance of La Paz-Iquique is 462 km) is about 0.0148 tons/km, whereas that of CRJ200 is 0.0129 tons/km. The CO2 emission in an LTO cycle of E190 is about 1.99 tons, whereas that of CRJ200 is 1.67 tons. Therefore, the change in aircraft type leads to the emission increase of these two routes. For Bogotá-Caracas, the weekly flight frequency in 2020 is 35, much higher than 14 in 2019. The rise of flight frequency results in its emission increase.

The routes with the most significant decline are São Paulo-Buenos Aires, Lima-Santiago de Chile, and São Paulo-Santiago de Chile. For all three routes, the emissions reductions were related to changes in the type and frequency of flights. In 2019, these three routes' average weekly flight frequency was 180, 160, and 140, whereas those in 2020 become 4, 9, and 14, respectively. In 2019, the leading aircraft types of São Paulo-Buenos Aires were 737-800, 320-232, 787-8, 777-300ER, and 777-200ER. In 2020, its primary aircraft will become 777-200ER and MD-11F. In 2019, Lima-Santiago de Chile’s main aircraft types were 787-9, 787-8, 320-233, 767-300ER, and 777-200ER. In 2020, its leading aircraft will become 320-214 and 787-9. In 2019, the top aircraft types of São Paulo-Santiago de Chile were 737-800, 777-300ER, 787-9, 767-300ER, and 777-300ER. In 2020, its primary aircraft will become 320-214 and 777-200ER. The significant change in flight frequency and aircraft types results in a substantial decline in emissions of these three routes.

### The impacts of the COVID-19 on the emissions of the airlines

**Figure 7:**
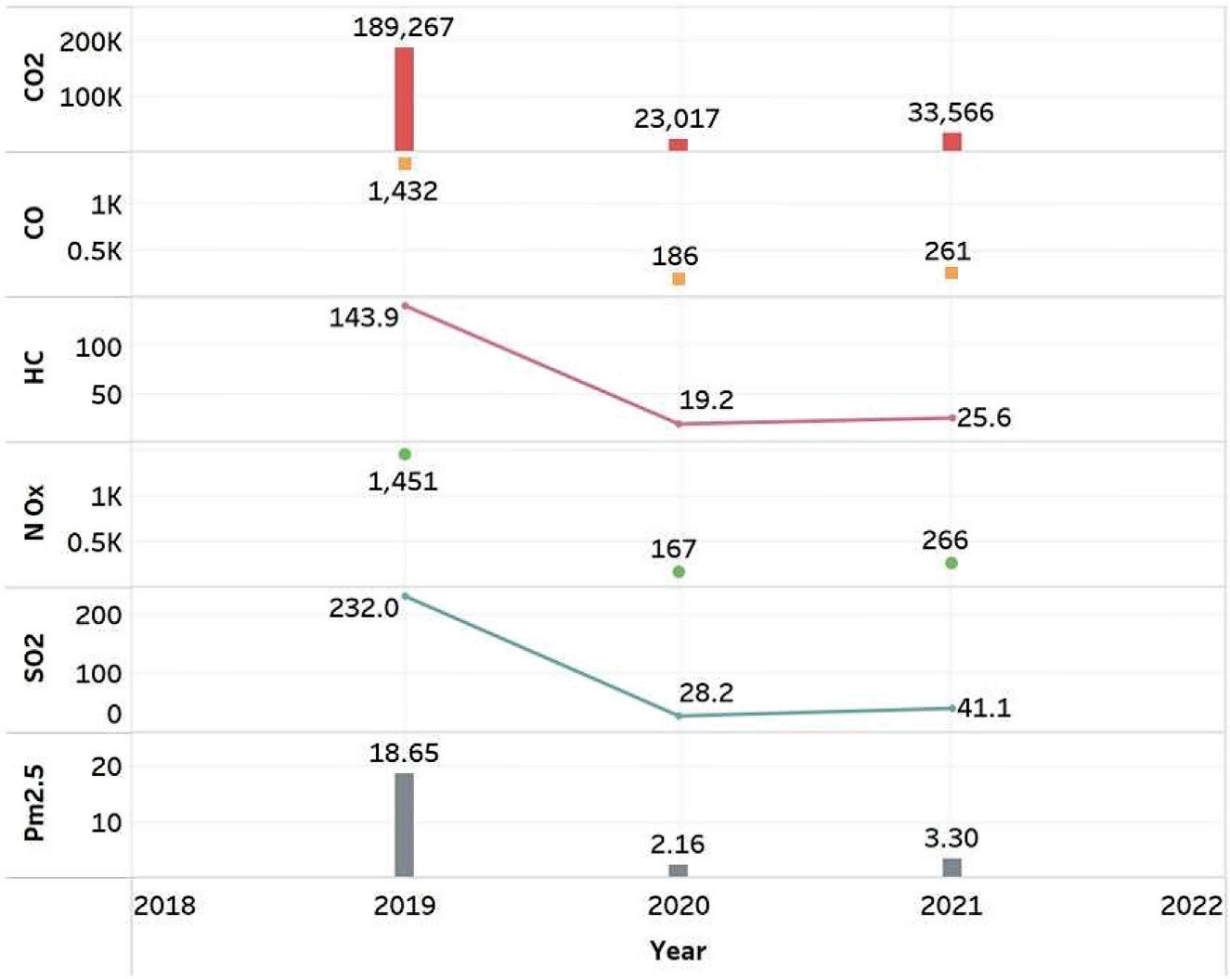
The average overall emissions of the airlines during 2019–2021 (tons)

First, we will discuss the impacts of COVID-19 on the average emission of airlines. The change in average overall emissions of the airlines during 2019–2021 is shown in Figure 7.

As shown in Figure 7, the average emissions of all airlines decreased significantly. The decline rates of CO_2_, CO, HC, NOx, SO_2_, and PM2.5 from 2019 to 2020 are 87.84%, 87.04%, 86.67%, 88.50%, 87.84%, and 88.40%, respectively. Consistent with the route results, there is still a big gap between 2021 and the level before the COVID-19. The average overall emissions of CO_2_, CO, HC, NOx, SO_2_, and PM2.5 in 2021 only account for about 17.74%, 18.22%, 17.80%, 18.31%, 17.74%, and 17.71%, respectively. From these data, it can be concluded that in 2021, the average emissions of the airlines in South America only recovered to less than 20% before the epidemic.

**Figure 8:**
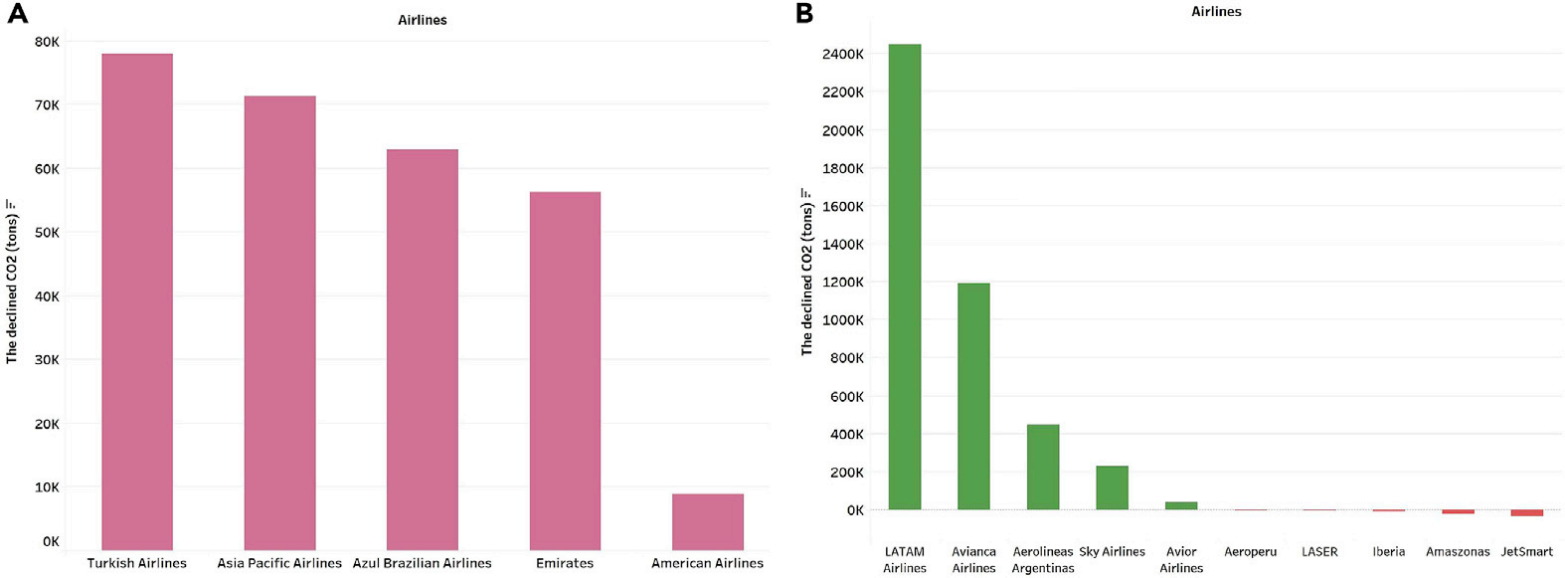
The airlines with the largest decline (A) The airlines with flights in 2019 and 2021 but few flights in 2020. (B) The airlines with flights in these three years.

Next, we will analyze the impacts of the COVID-19 on specific airlines. As with routes, we have divided airlines into two categories: the airlines with flights in 2019 and 2021 but few flights in 2020 and the the airlines with flights in these three years. For the former, we summarize the five airlines with the most significant emissions in 2019, as shown in Figure 8A. For the latter, we summarize the five airlines with the most significant decline and the airlines with the largest increase in 2020 compared with 2019, as shown in Figure 8B.

From Figure 8A, we can conclude that the five airlines with the most significant decline are Turkish Airlines, Asia Pacific Airlines, Azul Brazilian, Emirates, and American Airlines. For Turkish Airlines, according to its statistics, Central and South America account for about 1.1% of Turkish Airlines' international passenger traffic and 2.3% of passenger revenue (Turkish Airlines, 2022). However, Turkish Airlines significantly reduced its flights to the Americas due to limited government support for the airline industry during the COVID-19 pandemic in South America. At the same time, its airline revenues in the Americas and Eastern Europe also declined significantly, from $268 million in 2019 to $115 million in 2020. Among the five airlines, South American airlines and airlines from other countries indicate that uniform and strict immigration policies affect all airlines to some extent.

Figure 8B shows that the five airlines with the most significant decline are LATAM Airlines, Avianca Airlines, Aerolineas Argentinas, Sky Airlines, and Avior Airlines. Among the international routes in South America, LATAM Airlines had the most considerable reduction in emissions. LATAM Airlines cut 12,600 jobs in 2020, nearly 30% of its prepandemic workforce. It posted a net loss of $890 million in the second quarter of 2020 and filed for bankruptcy protection in New York in May 2020. Many airlines in South America have all been hit hard by the coronavirus outbreak, which has led to travel bans and a reluctance to fly.

However, some airlines’ emissions increased in 2020 compared with 2019. As shown in Figure 8B, the overall emissions of JetSmart, Amaszonas, Iberia, LASER, and Aeroperu have increased from 2019 to 2020. In other words, the international routes of these airlines in South America were not affected by the epidemic but increased the frequency of flights and updated aircraft types. For example, in 2019, JetSmart took Santiago de Chile as the base, and it operated two main routes to Peru: Santiago-Lima and Santiago-Arequipa. As a result, the total flight frequency in 2019 was about 1,300, and the average flying distance was 2,175.5 km. However, in 2020, although the aircraft is still the A320 series, JetSmart has expanded its service to Colombia and Brazil, and the total number of routes increased to 8. As a result, the flight frequency in 2020 increased to about 1,404, and the average flying distance became 3,031.88 km. The increased flight frequency, routes, and flying distance lead to these airlines’ emissions from 2019 to 2020.

## Discussion

This study focuses on exploring the impacts of COVID-19 on the aircraft emissions of the international routes in South America. The emissions in 2019 are considered as the data before COVID-19, and those in 2020 and 2021 are the data under COVID-19. First, we collect the flight information (including aircraft types, flight frequency, airline, flying distance, flying time, etc.) of all the international routes in South America and then calculate the overall emissions for each route and airline containing CO_2_, CO, HC, NOx, SO_2_, and PM2.5. The overall emissions include CCD emissions and LTO emissions. The CCD emissions are calculated through the modified BFFM2-FOA-FPM method, and the LTO emissions are calculated based on the ICAO standard method. Accounting for the emissions can better summarize the impact of aircraft activities on the environment and provide data and method references for putting forward corresponding countermeasures.

The main contribution of this paper to the literature is reflected in the following aspects. Firstly, this is the first attempt to analyze the impacts of COVID-19 on aircraft emissions. COVID-19 affects every aspect of the airline industry—operations, profits, revenues—but few previous studies have focused on its impact on emissions, and our work can fill this gap. Secondly, this is the first attempt to combine CO_2_ and non-CO_2_ emissions calculations. Some papers have focused on calculating CO_2_ and non-CO_2_ emissions, but there is still no unified method to combine the two accounting methods. Furthermore, the calculation method of CO_2_ emissions has not considered the difference of subseries and that of non-CO_2_ emissions has not focused on the emissions in the CCD stage. Therefore, we built a new method—modified BFFM2-FOA-FPM method—to calculate the CCD stage’s CO_2_ and non-CO_2_ emissions. The LTO emissions are calculated using the International Civil Aviation Organization (ICAO) standard procedure. The calculated results' error rate ranges from 1.09% to 9.14%. This paper applies the method for international routes in South America, but it can also be used to account for other countries or regions. Thirdly, this is the first time calculating the aircraft types' emission intensity. We divide the route distance into 11 groups: 0–500 km; 501–1,000 km; 1,001–1,500 km; 1,501–2,000 km; 2,001–2,500 km; 2,501–3,000 km; 3,501–3,500 km; 3,501–4,000 km; 4,001–4,500 km; 4,501–5,000 km; and 5,001–5,500 km. Then we get the six pollutions of aircraft types at these different distances to ensure that the calculation results are accurate. Compared with existing research, our results cover more detailed aircraft types, and we corrected the results with the actual flight time to make it more accurate.

The main conclusions and policy recommendations are as follows: first, due to the COVID-19 pandemic, the number of routes and average distances of international routes in South America in 2020 were much lower than in 2019. Second, affected by the epidemic, regional aircrafts such as E190 and CRJ200 were widely used on international routes in South America in 2020. Third, influenced by the COVID-19, the overall emission in 2020 decreased significantly compared with that in 2019, increased slightly in 2021, but did not return to the emission before the epidemic. Furthermore, CCD emissions accounted for the lowest proportion of total emissions in 2020. Fourth, among these six pollutions, CO_2_ is much higher than that of another one, so the primary emissions from aviation are still CO_2_. Fifth, the average emissions of the routes and airlines decreased significantly due to the pandemic, but the impacts on specific airlines significantly differ. Some airlines’ emissions increased in 2020 compared with 2019, such as JetSmart, Amaszonas, Iberia, LASER, and Aeroperu. The increased flight frequency and updated aircraft types result in their reverse growth in emissions.

The technological and market paths are generally considered the most probable approaches to controlling aircraft emissions (Cui and Li, 2017). The technical method mainly includes using more efficient engines, introducing biomass fuel, etc. The market path primarily consists of pollution taxes, etc. Calculating CO_2_ and non-CO_2_ emissions is the basis and prerequisite for levying pollution taxes, so the work of this article has crucial significance.

### Limitations of the study

Several limitations exist in this study. First, it should be noted that the overall emissions are calculated through the standard LTO stage. Therefore, the emissions due to delays are not considered, and the aircraft transfer caused by temporary weather is not considered. Second, although this study has calculated the aircraft emissions in South America under the influence of COVID-19, the current situation of the global COVID-19 is complex. Therefore, it is necessary to conduct more research such as relevant predictions based on the data in this paper to explore the sustainable development of the global aviation industry under the background of the future epidemic to provide valuable insights for the formulation of more appropriate emission reduction policies. Furthermore, this study has not evaluated the emissions from air cargo. Therefore, further investigation can calculate the emissions caused by delay, aircraft transfer, and air cargo.

## STAR★Methods

### Key resources table

**Table:** 

REAGENT or RESOURCE	SOURCE	IDENTIFIER
**Deposited data**		

Route origin and destination data	VariFlight (2022)	https://www.variflight.com/
Flight data	VariFlight (2022)	https://www.variflight.com/
Engines of each aircraft	EASA (2022)	https://www.easa.europa.eu/domains/environment/icao-aircraft-engine-emissions-databank

### Resource availability

#### Lead contact

Further information and requests should be directed to the lead author, Qiang Cui (cuiqiang@seu.edu.cn).

#### Materials availability

This study did not generate new unique materials.

### Method details

#### Modified BFFM2-FOA-FPM method

The CCD emissions *E*(*Q*) can be calculated by

(Equation 1) $$\begin{array}{c} E_{j}\left(Q\right)=I_{j}*F\left(Q\right)=I_{j}*M_{fuel}*weight\left(Q\right)=I_{j}*\left(1-M_{ff}\right)*weight\left(Q\right)\\ \;=I_{j}*\left(1-\prod_{i=1}^{n}\frac{W_{i}}{W_{i-1}}\right)*weight\left(Q\right)=I_{j}*\left[1-e^{-\frac{dis*ratio_{cr}}{10*v}}\right]*weight\left(Q\right)\\ \;=I_{j}*\left[1-e^{-\frac{dis*ratio_{cr}}{10*v}}\right]*(aircraftbareweight+100*\\ \;\;\left(load\;factor*number\;of\;seats\right)+50*seat) \end{array}$$

These notations come from the Base of Aircraft Data (Eurocontrol, 2004). *I*_*j*_ is the emission coefficient of pollution j of aviation kerosene (EASA, 2022). *weight* (*Q*) is the total weight of the aircraft.*M*_*fuel*_ is the fuel coefficient, $M_{ff}=\prod_{i=1}^{n}\frac{W_{i}}{W_{i-1}}\;$ is a fuel weight proportionality coefficient, which is usually calculated by Fuel Percentage Method (FPM) (Cui, 2019). The total sections of a whole flight contain seven task sections: Engine Starting, Taxiing, Taking Off, Climbing, Cruising, Descending and Landing. $W_{i}/W_{i-1}$ aswq the fuel weight proportionality coefficient of task section *i*(*i* = 1,2, …,7). *number of seats* is certified seat number, *seat* is the actual passenger number.

As we only consider the CCD section in this study, so we define the $W_{i}/W_{i-1}$ of other sections is 1. The $W_{i}/W_{i-1}$ of Climbing and Descending are 0.980 and 0.990. The equation of the CCD section to calculate $W_{i}/W_{i-1}$ is $W_{i}/W_{i-1}=e^{-\frac{dis*c_{cr}}{10*v*LD_{cr}}}$. *dis* is the cruising distance, *v* is the cruising speed, *c*_*cr*_ is the fuel consumption ratio when the aircraft is cruising, *LD*_*cr*_ is the lift-drag ratio when the aircraft is cruising. The value of *c*_*cr*_ and *LD*_*cr*_has direct relationships with the aircraft type. We define $ratio_{cr}=\frac{c_{cr}}{LDc_{cr}}$, and then for the cruising task section, the $W_{i}/W_{i-1}$ is. $W_{i}/W_{i-1}=e^{-\frac{dis*ratio_{cr}}{10*v}}\text{.}$

The actual flying time of each flight is applied to check the results of *ratio*_*cr*_, and get the emission intensity.

For CO_2_, the emission coefficient is fixed, which is *I*_*CO*2_ = 3.157 *kg*/*kg*;

For SO_2_, the emission coefficient is fixed, which is *I*_*SO*2_ = 3.870 *kg*/*kg*;

For CO and HC, $I_{j}=I_{j0}*\frac{\theta^{3.3}}{\delta^{1.02}}\text{.}$ *θ* is the ratio of outside temperature to 288 K; *δ* is ratio of external pressure to sea level pressure. *I*_*j*0_ is the standard emission coefficient of an LTO stage OF CO or HC (g/kg).

For NOx, $I_{NOx}=I_{j0}*\frac{\delta^{0.51}}{\theta^{1.65}}*\text{exp}\left(19.0*\left(0.0063-\frac{0.622*\varphi *Pv}{P-\varphi *Pv}\right)\right)$. *I*_*j*0_ is the standard emission coefficient of an LTO stage of NOx (g/kg). *θ* is the ratio of outside temperature to 288 K; *δ* is ratio of external pressure to sea level pressure.*φ* is atmospheric relative humidity;*P* is external pressure; *Pv* is atmospheric saturation pressure, which is calculated by Goff-Gratch formula (Detwiler, 1983):

$$\mathrm{lg}Pv=10.79574*\left(1-\frac{273.16}{T}\right)-5.02800*\mathrm{lg}\left(\frac{T}{273.16}\right)+1.50475*10^{-4}*\left[1-10^{8.2969*\left(1-\frac{T}{273.16}\right)}\right]+0.42873*10^{-3}*\left[10^{4.76955*\left(1-\frac{T}{273.16}\right)}\right]+0.78614$$

According to relevant physical laws (Smith et al., 1970; Abu-Ghannam and Shaw, 1980; Erhardt and Mecklenburg, 1994), the external pressure *P* is $\text{P}=101325\text{∗}{\left(1-\frac{H}{44300}\right)}^{5.256}\text{.}$ H is height. The outside temperature T is $\text{T}=291.15-\frac{6*H}{1000}\text{.}$ The atmospheric relative humidity *φ* is $\text{φ}=100\text{∗}\frac{a*\left(1+T/273.16\right)}{0.8*Pv}$. *a* is absolute humidity, and it is. $\text{a}=\frac{26}{233211}*T^{3}-\frac{302}{3731}*T^{2}+\frac{569}{29}*T-\frac{17461}{11}\text{.}$

For PM2.5, according to First Order Approximation (FOA) method (Wasiuk et al., 2016), it can be divided into Nonvolatile Component Fine Particles (NCFP) and Volatile Component Fine Particles (VCFP). For NCFP, $I_{NCFP}=0.054*AFR*{\left(SN\right)}^{1.234}+0.877\text{.}$ The unit of *I*_*NCFP*_ is mg/kg. *AFR* is Air-Fuel Ratio, which is decided by height. *SN* is engine smoke, which can be found in the ICAO Aircraft Engine Emissions Databank (EASA, 2022). For VCFP, it contains Volatile Organic Components (VOC) and Volatile Sulfur Components (VSC). For VOC, $I_{VOC}=\sigma *I_{HC}\text{.}$. *σ* is the ratio of VOC to the emission coefficient of HC, which can be found in ICAO Aircraft Engine Emissions Databank (EASA, 2022). For VSC, $I_{VSC}=3*10^{6}*0.2\%*3.3\%\text{.}$ 0.2% is fuel sulfur content and 3.3% is sulfur conversion coefficient. Therefore, for PM2.5, *I*_*PM*2_._5_ = *I*_*NCFP*_ + *I*_*VOC*_ + *I*_*VSC*_.

#### ICAO standard method to calculate LTO emissions

The LTO stage includes approaching, taxiing, taking-off, and climbing, which defines climbing as the boundary layer from the end of aircraft takeoff to the aircraft’s flight out of the atmosphere. Therefore, this paper uses the standard LTO cycle definition specified by ICAO to calculate the fuel consumption, including all activities at an altitude below 3000 feet (915m) near the airport. Therefore, this stage is not directly related to the route. The calculation formula of the five non-CO_2_ pollution emissions in the LTO stage is:

(Equation 2) $$E_{LTO}=\sum_{m}P_{a}*N_{a}*C_{m}*t_{m}$$

*E*_*LTO*_ is the emissions in the LTO stage; *P*_*a*_ is the standard emissions of the engine of aircraft type *a* (unit: kg); *N*_*a*_ is the number of engines of aircraft type *a*;*C*_*m*_ is the thrust setting of stage *m*; *t*_*m*_ is the working time of phase *m*. The value range of *m* is 1, 2, 3, and 4, respectively corresponding to the four stages of takeoff and landing in the aircraft flight process: takeoff, climb, approach and taxiing (Xue et al., 2021). According to the standard LTO cycles defined by ICAO, when the aircraft is taking off, its engines are at 100% thrust and working time is 0.7 min; when the aircraft is climbing, its engines are at 85% thrust and working time is 2.2 min; when the aircraft is approaching, its engines are at 30% thrust and working time is 4 min; when the aircraft is taxiing, its engines are at 7% thrust and working time is 26 min. Therefore, in a standard LTO cycle, the total working time is 32.9 min.

The fuel consumption rate is calculated as:

(Equation 3) $$F_{am}=\frac{1}{A}\sum_{j}K_{j}*F_{jmi}$$

*A* is the total number of airlines with aircraft type *a*; *j* is the type of engine of the aircraft; *K*_*j*_ is the number of aircraft type *a* equipped with engine type *j*; *F*_*jmi*_ is the fuel consumption rate of engine type *j* under the *m* setting. The data is from the ICAO Aircraft Engine Emissions Databank (EASA, 2022). This formula is based on the weighted average of all possible engine types of the domestic routes in China.

## Data Availability

• This paper analyzes existing, publicly available data which are listed in the Key resources table. The specific steps of data collection can be found in Table S1. The CCD and LTO emissions of each route and airline for the six pollutions are shown in Tables S2, S3 and S4. • The emission intensities of the six pollutions of each aircraft type can be found in Tables S5, S6 and S7. • This paper does not use code. • Any additional information required to reanalyze the data reported in this paper is available from the lead contact upon request.

## Supplemental information

Table S1. Specific steps of data collection, related to STAR Methods

Table S2. The emissions of six pollutions in 2019, related to STAR Methods

Table S3. The emissions of six pollutions in 2020, related to STAR Methods

Table S4. The emissions of six pollutions in 2021, related to STAR Methods

Table S5. The emission intensities of the six pollutions in 2019, related to STAR Methods

Table S6. The emission intensities of the six pollutions in 2020, related to STAR Methods

Table S7. The emission intensities of the six pollutions in 2021, related to STAR Methods
